# Is air pollution a risk factor for rheumatoid arthritis?

**DOI:** 10.1186/s12950-015-0092-1

**Published:** 2015-07-30

**Authors:** Mickael Essouma, Jean Jacques N. Noubiap

**Affiliations:** Division of Medicine, Sangmelima’s Reference Hospital, P.O. Box 890, Sangmelima, Cameroon; Department of Medicine, Groote Schuur Hospital and University of Cape Town, Cape Town, South Africa; Medical Diagnostic Center, Yaoundé, Cameroon

**Keywords:** Rheumatoid arthritis, Pathogenesis, Environmental risk factors, Air pollution

## Abstract

Rheumatoid arthritis is a chronic inflammatory debilitating disease triggered by a complex interaction involving genetic and environmental factors. Active smoking and occupational exposures such as silica increase its risk, suggesting that initial inflammation and generation of rheumatoid arthritis-related autoantibodies in the lungs may precede the clinical disease. This hypothesis paved the way to epidemiological studies investigating air pollution as a potential determinant of rheumatoid arthritis. Studies designed for epidemiology of rheumatoid arthritis found a link between traffic, a surrogate of air pollution, and this disease. Furthermore, a small case–control study recently found an association between wood smoke exposure and anticyclic citrullinated protein/peptide antibody in sera of patients presenting wood-smoke-related chronic obstructive pulmonary disease. However, reports addressing impact of specific pollutants on rheumatoid arthritis incidence and severity across populations are somewhat conflicting. In addition to the link reported between other systemic autoimmune rheumatic diseases and particulate matters/gaseous pollutants, experimental observation of exacerbated rheumatoid arthritis incidence and severity in mice models of collagen-induced arthritis after diesel exhaust particles exposure as well as hypovitaminosis D-related autoimmunity can help understand the role of air pollution in rheumatoid arthritis. All these considerations highlight the necessity to extend high quality epidemiological researches investigating different sources of atmospheric pollution across populations and particularly in low-and-middle countries, in order to further explore the biological plausibility of air pollution’s effect in the pathogenesis of rheumatoid arthritis. This should be attempted to better inform policies aiming to reduce the burden of rheumatoid arthritis.

## Background

Rheumatoid arthritis (RA) is a systemic autoimmune disease that primarily targets joints leading to progressive joint erosions, and which affects 0.24 % [95 % confidence interval (CI) 0.23 to 0.25 %] of the world population. RA patients continue to face steep rises of the burden of disease, with disability-adjusted life years estimates increased from 3 335 000 (95 % CI 2 573 000 to 4 192 000) in 1990 to 4 815 000 (95 % CI 3 705 000 to 6 056 000) in 2010 [1, 2]. Based on serologic features, two forms are identified: seronegative and seropositive RA [3]. Hereditability of the disease accounts for 50–60 % of its variance, the HLA-DRB1 shared epitope–containing allele being the strongest genetic risk factor [4–6]. Some environmental risk factors are playing out on this genetic background to determine the occurrence of RA. Besides active and heavy tobacco smoking which is the most important environmental risk factor for RA with its attributable risk sustained for up to 20 years after discontinuation, other potential risk factors include estrogens, low socioeconomic status, silica dust, abestoses, mineral oil, diet and infections [5, 6]. However, the exact etiopathogenesis of RA is still not clearly elucidated.

Air pollution, a mixture of suspended particulate matters (PM) of different diameters (<10 μm [PM_10_], <2.5 μm [PM_2.5_] and <0.1 μm [PM_0.1_]) and gases (nitrates [NO_2_], sulphur dioxide [SO_2_], ozone [O_3_], and carbon monoxide) has recently been paid more attention in the field of RA [7]. Main sources include: traffic, industry, stationary fuel burners, forest fires, and solid fuel combustion [7]. Tropospheric pollutants drive climate change and adverse health effects including chronic obstructive pulmonary disease (COPD), cancers, cataracts, stillbirths, and cardiovascular diseases [7].

Assuming that active smoking and occupational exposures increase the risk of RA, it was suggested that initial inflammation and production of RA-related autoantibodies in the lungs may lead to RA [4]. This idea together with the inflammation-related burden of disease attributable to atmospheric pollution guided epidemiological studies of air pollution in relation to RA, in view of eventual preventive strategies.

### A link between sources of air pollution and rheumatoid arthritis

Currently, the association between sources of atmospheric pollution and RA has been investigated in three large epidemiological studies: the Nurses’ Health Study (NHS) in the United States [8, 9], the British Colombian (BC) study in Canada[10], and the Swedish Epidemiological Investigation of Rheumatoid Arthritis (EIRA) [11, 12]. In the NHS, association of distance to road-a marker of traffic pollution exposure- and incidence of RA was studied in 90297 women. After adjustment for multiple confounders (age, calendar year, race, cigarette smoking, parity, lactation, menopausal status and hormone use, oral contraceptive use, body mass index, physical activity, and census-tract-level median income and house value), women living within 50 m of a road had an incremented risk of RA compared with those living 200 m or farther away (hazard ratio [HR] = 1.31; 95 % CI 0.98–1.74) [8]. This finding was more consistent among nonsmokers (HR = 1.62; 95 % CI, 1.04–2.52). Similarly, the BC study assessed the effects of proximity to traffic, ambient air pollution, and community noise on the risk of developing RA using health records of 640041 subjects at risk of developing RA in the Border Air Quality Study cohort. Residential proximity to traffic five years prior to RA diagnosis, but consistently not noise, was associated with an increased risk of RA; with highest risks noted for highways and with greater proximity. Since health service records did not contain informations on low socioeconomic status, nonwhite race, and smoking which are all risk factors for RA highly prevalent close to roadways, the authors did not adjust for these factors in statistical analyses. Nevertheless, there was little confounding due to neighborhood-level income, and smoking was unlikely to be an important confounder according to sensitivity analyses [10]. Additionally, Sigari et al. [13] recently found significantly elevated anticyclic citrullinated protein/peptide antibody (ACPA) levels in 56 wood smoke-induced COPD patients compared to 56 tobacco-induced COPD patients, and to 56 healthy controls. It is noteworthy that ACPA (the most specific biological markers with predictive and prognostic value in RA patients) are elevated in patients’ sera five to 10 years prior to diagnosis with RA [4, 11, 12, 14], suggesting that factors initiating autoimmunity in RA may act before appearance of symptoms and signs characteristic of clinical disease. In this sense, wood smoke might be a risk factor for RA. However, tropospheric pollutants including PM_10,_ PM_2.5,_ SO_2_, and NO_2_ were associated with RA neither in the NHS [9], nor in the BC study [10]. In the same way, the EIRA Swedish population-based study of 1497 incident RA patients compared with 2536 age and sex matched controls investigated the impact of an interquartile range increase (2 μg/m^3^ for PM_10_, 8 μg/m^3^ for SO_2_, and 9 μg/m^3^ for NO_2_) in each pollutant from traffic and home heating sources in the 5th, 10th, and 20th years prior to symptom onset (considering increment of ACPA titers five to 10 years before occurrence of clinical signs and symptoms of RA) and average exposure on the risk of all RA and the risk of RA serologic features. Total RA risks were increased for exposure to the gaseous pollutants (NO_2_ and SO_2_) in the 10th year before onset, but were no more statistically significant after adjustment for smoking and education (odds ratio [OR] =1.18, 95 % CI 0.97–1.43] and OR = 1.09, 95 % CI: 0.99–1.19) for SO_2_ and NO_2_ respectively). Stronger elevated risks at the same time point were noted for ACPA-negative RA cases even after adjustment for smoking and education (OR = 1.48, 95 % CI 1.13–1.95 and OR = 1.22, 95 % CI 1.07–1.40 for SO_2_ and NO_2_ respectively) [12]. Discrepant results about NO_2_ and SO_2_ between studies indicate the necessity to better clarify their role in the pathogenesis of RA [8, 10, 12], as well as that of PM which can be formed from these gases by enucleation in the air [7], and considering the high correlation between NO_2_ and PM_10_ [12]. Furthermore, results have not been verified across populations including different age groups, especially the ageing population which is more at risk of developing RA [1], and mixed ancestries. Moreover, a major shortcoming of the study by Hart el al. [9] which examined the association between specific pollutants and incident RA within the NHS was the lack of informations necessary for assessment of the impact of each nurses’ total air pollution exposure experience on the incidence of RA (amount of time spent at each home address, exposure to pollutants at locations other than the residence, exposure models that can perfectly predict personal exposures) [9]. In addition, the follow up period of the BC cohort was probably shorter than the induction period of tropospheric pollutants [10]. Taken together, sources of air pollution including traffic and probably solid fuels are associated with RA, but smoking may attenuate this association. Yet, inconclusiveness persists regarding the role of tropospheric pollutants including NO_2_, SO_2_ and PM. Indirect arguments can help support the role of these pollutants in the pathogenesis of RA.

### Indirect evidence for a potential link between air pollution and rheumatoid arthritis

Despite sparse literature on air pollution and RA per se, tropospheric pollutants have repeatedly been associated with various systemic autoimmune rheumatic diseases (SARDs) [15–18]. In particular, PM_2.5_ levels 24 h–48 h before visits were significantly associated with anti-double stranded DNA serum specific autoantibodies in 237 Canadian patients followed up for systemic lupus erythematosus (SLE) during a time frame of seven years [15]. Likewise, a Brazilian study reported a link between SO_2_ exposures 14 days earlier and increased hospital admissions for pediatric autoimmune rheumatic diseases [16]. Moreover, Bernatsky et al*.* recently studied associations between local-scale ambient NO_2_ and PM_2.5_ in Calgary, Alberta-Canada, using Land Use Regression models (a preferred approach for exposure estimation including individual exposure estimation) and SARDs (SLE, Sjogren’s Syndrom, scleroderma, polymyositis, dermatomyositis, or undifferentiated connective tissue disease) [17]. Resultantly, ambient PM_2.5_ levels but not NO_2_ are strongly associated with SARDs in Alberta (OR adjusted for age, sex and income = 1.10, 95 % CI 1.01–1.22).

Based on the aforementioned studies and the small amount of data addressing air pollution in relation to RA together with extrapolations from relevant data in other areas, RA could be associated with PM, but also NO_2,_ SO_2_ and O_3_ exposures as well. Indeed, PM pollutants are the most toxic tropospheric pollutants for all-cause morbidity and mortality associated with air pollutants [19–22]. In addition, they have been associated with other SARDs [15, 17]. Still, they can interact with gaseous pollutants that have been associated with RA [11], and even result from their transformation [22]. Most importantly, exposure to diesel exhaust particles (nano PM) during arthritis development exacerbated incidence and severity of RA in mice models of collagen-induced arthritis [23, 24]. However, NO_2_ and SO_2_ linked with RA in the EIRA study [11], and O_3_ linked with risk of RA in the BC study [10] all appear less toxic than PM and their respective concentrations highly depend on their complex interactions. Furthermore, NO_2_ may essentially serve as a proxy for PM, and O_3_ increases permeability of the respiratory epithelium to PM, hence exacerbating their actions [22].

Reports on the precise mechanistic link between these specific air pollutants and RA are somewhat conflicting. Anyhow, extrapolating from studies on air pollution and respiratory diseases [21, 22] as well as studies on air pollution and other SARDs [15–17] together with knowledge on mechanism of action of tropospheric pollutants [15–24], local lung and systemic oxidative stress and inflammation may be the central underlying mechanism [4, 18, 19, 25, 26]. In particular, free reactive oxygen species released by fine/ultrafine PM or gaseous pollutants inhaled in the respiratory tract are capable of activating nuclear factor ƙappa B (NF-ƙB), a key regulator for pro-inflammatory cytokine production in RA patients [18, 19, 25], leading to excess T helper lymphocyte type1 (Th1) production of tumor necrosis factor alpha, interleukin-1 and interleukin-6 [25]. These cytokines stimulate resting monocytes to mature dendritic cells which then present autoantigens, co-stimulating self-reactive T lymphocytes that migrate to target tissues (preferentially synovial joints), and cause destruction of cells expressing autoantigens [18]. Hence, there is persistent amplified chronic inflammation, a major prerequisite for protein bounded arginine citrullination and subsequent ACPA production that will later on lead to clinical signs of RA in genetically susceptible individuals [4, 26]. Moreover, reactive oxygen species per se are capable of worsening RA through induction of periarticular bone erosion [26].

Beyond oxidative stress, air pollution might further contribute to RA development and exacerbation through hypovitaminosis D [27–30]. Remarkably, the link between air pollution and low serum vitamin D has been investigated in astonishingly few cross-sectional studies [31–33]. Agarwal et al. [31] compared serum total 25-hydroxycholecalciferol (25-OH-D)-the best indicator of vitamin D storage- of 34 infants (aged 9–24 months) and toddlers in Mori Gate–a highly polluted area- with serum total 25-OH-D of a similar number of age and sex matched controls living in Gurgaon, a less polluted area. Mori Gate inhabitants had significantly lower mean serum 25-OH-D compared with Guargaon inhabitants (11.7 ± 7 ng/ml vs 27.1 ± 7 ng/ml, *p* < 0.001) [31]. Another study [32] evaluated the characteristics and prevalence of subjects with serum 25-OH- D less than 75 nmol/l among 85 postmenopausal women engaging in outdoor activities in either Brussels (urban highly polluted area) or the countryside (rural less polluted area) and recruited from the rheumatology outpatient clinic. Despite a higher sun exposure index (113 vs. 87, *P* < 0.001), city dwellers had a higher prevalence of serum 25-OH- D less than 75 nmol/l (84 % vs. 38 %) when compared with rural residents. Furthermore, Hosseinpanah et al. [33] studied the role of air population on serum total 25-OH-D in 200 healthy middle aged Iranian women divided into two groups with respect to the level of pollution in their residence: 100 women living in Tehran (highly polluted) and 100 women living in Ghazvin. After adjustment for important determinants of vitamin D status including age, body mass index, daily dietary vitamin D, calcium, phosphorus, protein, and the levels of sunlight exposure, living in higher polluted area was associated with lower levels of serum 25-OH-D [33]. Particularly relevant here, ~90 % of the body’s requisites for vitamin D are covered by skin synthesis in presence of sun’s ultraviolet B (UVB) radiations [33]. Also, 99 % of UVB radiations with wavelength 291–320 nm are absorbed by the ozone layer which is increased in situations of incremented air pollution levels [30]. Along with O_3_, NO_2_ and SO_2_ can absorb UVB in polluted atmosphere, thus reducing the effectiveness of sun exposure on skin synthesis of vitamin D [30]. Therefore, air pollution lowers serum vitamin D independently of sun exposure and other determinants of vitamin D status, probably through UVB radiations absorption by gaseous pollutants, and could thus increment RA incidence.

In fact, the active form of vitamin D, 1,25-dihydroxyvitamin D_3_ [1,25(OH)_2_D_3_], acts as an immunomodulator by binding to vitamin D receptor (VDR) besides its metabolic activities [27–30, 33]. Remarkably, 1,25(OH)_2_D_3_ is capable of inducing natural killer and T regulatory cells, thereby preventing autoimmunity [27]. Furthermore, 1,25 (OH)_2_D_3_ suppresses Th1 responses via interferon-γ inhibition, prevents monocyte differentiation into dendritic cells as well as antibody formation, and downregulates NF-ƙB [27].

**Fig. 1:**
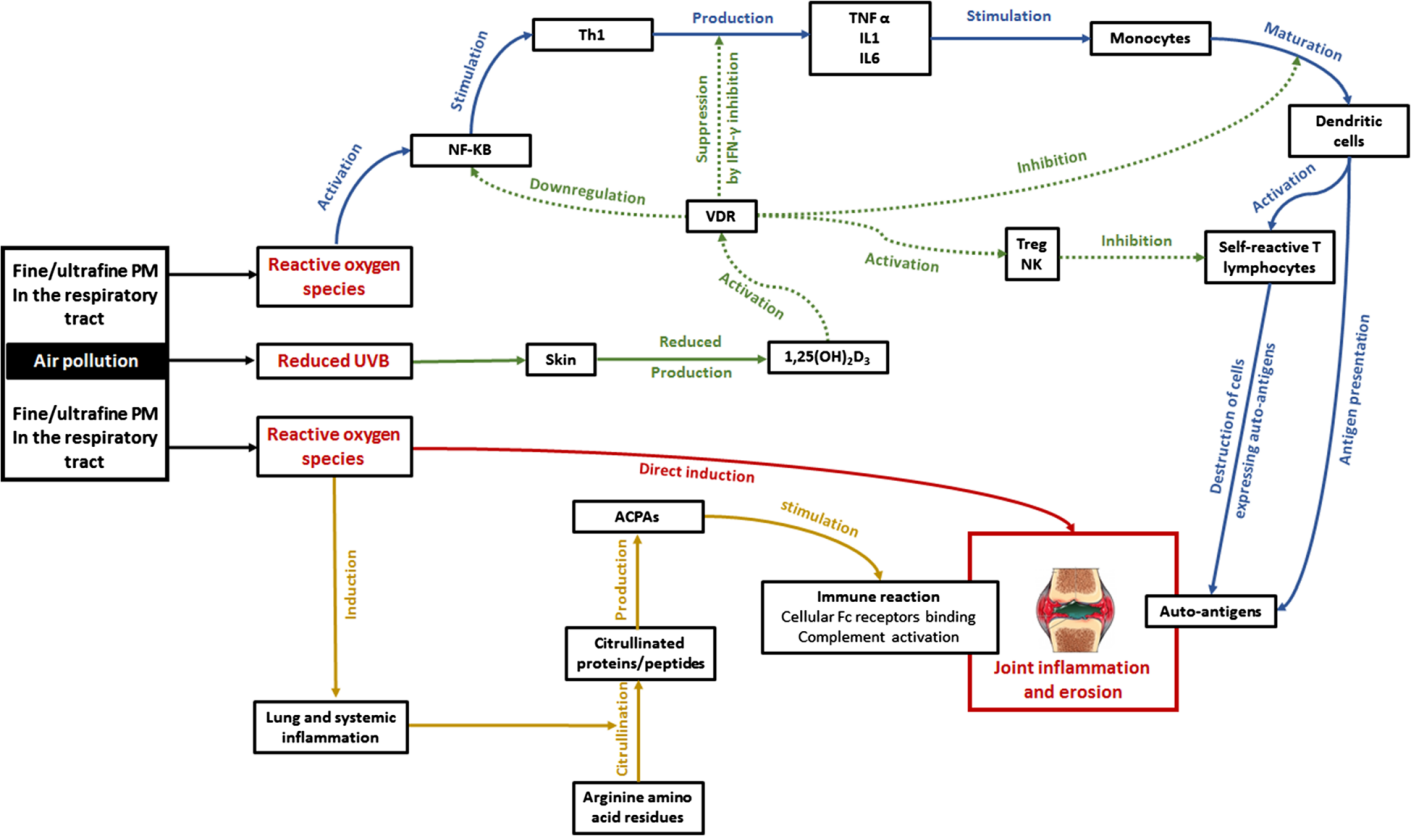
Schematic representation of mechanisms putatively influencing rheumatoid arthritis through air pollution exposure. Four main mechanistic pathways are represented (blue, brown, red, and green arrows). Blue arrows: free reactive oxygen species released by fine/ultrafine particulate matter (PM) inhaled in the respiratory activate nuclear factor ƙappa B (NF-ƙB) that stimulates the production of tumor necrosis factor alpha (TNF-α), interleukin 1 (IL1) and interleukin 6 (IL6) by T helper lymphocytes type 1 (Th1). These cytokines stimulate resting monocytes to mature dendritic cells which then present autoantigens, co-stimulating self-reactive T lymphocytes that migrate to target tissues (preferentially synovial joints), and cause destruction of cells expressing autoantigens and thereby joint inflammation and erosion. Brown arrows: free reactive oxygen species cause chronic lung and systemic inflammation that enhance citrullination of arginine amino acid residues into citrullinated proteins/peptides. These citrullinated products induce anticyclic citrullinated protein/peptide antibodies (ACPAs) production that will later on lead to immune reaction through binding to cellular Fc receptors and complement activation, and finally cause joint inflammation and erosion. Red arrow: reactive oxygen species per se can worsen joint inflammation and erosion. Green arrows: Reduced ultraviolet B (UVB) radiation levels lead to decreased skin synthesis of 1,25-dihydroxyvitamin D_3_ [1,25(OH)_2_D_3_] which acts as an immunomodulator through vitamin D receptor (VDR) activation. Resultantly, immunomodulatory activities of 1,25(OH)_2_D_3_ represented by green doted arrows are not met, and RA can develop. IFN-γ interferon gamma; NK natural killer cells; and Treg T regulatory cells

Altogether, local lung and systemic oxidative stress along with hypovitaminosis D may trigger RA in the context of air pollution (Fig. 1).

### Air pollution and rheumatoid arthritis with focus on low-and-middle income countries

Considering that majority of the world population resides in low-and-middle-income countries (LMICs), the number of affected people in this part of the world are projected to increase in the upcoming years. For instance, with an additional prevalence of 0.1 %, four million supplemental individuals might be affected [34]. Furthermore, a low socio-economic status has been associated with both increased risk of developing RA and increased mortality of RA patients [35]. Yet, RA is neglected in LMICs undoubtedly because of gaps in estimates of the burden of disease along with lack of communication strategies informing policy makers on the urgency of the problem [34].

As concerns sub-Saharan Africa, potential RA genetic background and environmental risk factors are quite different from those of other parts of the world despite an overall similar phenotype [4, 14, 36–38]. Notheworthy, smoking is not a risk factor for RA in this population [14, 36]. Hence, RA in Black Africans may be determined by specific genetic and environmental factors such as lower socio-economic status, and infections [4, 14, 37]. But the role of infections is controversial [14, 36], with parasitic infections suggested to protect against RA in Sub-Saharan Africa since they rage at endemic levels in that area [14, 39, 40]. Admittedly, parasites cannot resist in a milieu characterized by amplified immuno-inflammatory activation. Subsequently, the protection against RA is probably a consequence of defense mechanisms developed by parasites to survive in the host organism [39, 40]. Along this line, ES-62, a glycoprotein secreted by a filarial nematode called Acanthocheilonema viteae has been shown to restore interleukin-10 producing regulatory B cells (Breg) in collagen-induced arthritis mice models [39]. It is notable that IL-10 is an anti-inflammatory cytokine associated with downregulation of Th1/Th17 responses [39]. Therefore, it appears necessary to pursue research of risk factors for RA in sub-Saharan Africa, and air pollution could be a plausible, if not the most important candidate.

Household air pollution (HAP) defined as “air pollution generated by household fuel combustion, leading to indoor air pollution, and contributing to ambient air pollution” [41] is mostly prevalent in LMICs where nearly three million people still rely on solid fuels for heating, cooking and lighting [21, 41–43] resulting in approximately 4.3 million premature deaths worldwide [41]. Still, peak prevalences of HAP exposure worldwide occur in sub-Saharan Africa (77 % with the number of affected individuals almost doubled from 333 million to 646 million over three decades) and Southeast Asia (61 %) [21]. Of note, this prevalence also varies within countries, with up to 100 % households concerned in some rural areas [44]. Moreover, individuals with a lower socioeconomic status have an incremented risk of exposure to atmospheric pollution compared with individuals who have high incomes (differential exposure) [11, 45, 46]. Likewise, people with a lower socioeconomic status are at increased risk of developing air pollution-related health hazards compared with those who have a higher socioeconomic status (differential susceptibility) [11, 45, 46]. Taken collectively, poverty increases the risk of air pollution and exacerbates its consequences. Hence, Africa and Southeast Asia may carry the heaviest burden of air pollution.

RA has been associated with a lower socioeconomic status [5, 35] which may be a marker for residential exposure to air pollution [11]. Indeed, it is a confounder of the association between tropospheric pollutants and RA [9, 11]. In this light, results from the NHS which failed to demonstrate any association between RA and specific pollutants [9] are unlikely to be generalized in impoverished societies given the high economic status of the included nurses thus residing in less polluted areas [9], unlike populations from LMICs.

In total, the burden of RA and increased atmospheric pollution coincide in LMICs-especially in sub-Saharan Africa and Southeast Asia- with poverty raising the risk of air pollution and exacerbating air pollution. However, there is still a small amount of data on air pollution and RA coming from LMICs.

## Conclusion

Traffic, a surrogate of air pollution, has been associated with incident RA. Whether this environmental factor is causally associated with RA across populations is still a matter of debate. Based on indirect arguments, as well as exacerbation of RA incidence and severity in mice models of collagen-induced arthritis after diesel exhaust particles exposure, local lung and systemic oxidative stress together with vitamin D deficiency-related autoimmunity may help to clarify a putative mechanistic link between tropospheric pollutants and RA. Additional well-designed epidemiological studies investigating different sources of air pollution and specific pollutants in relation to RA across populations, especially in LMICs are thus needed to better explore the biological plausibility of air pollutions’ effects in RA. If a causal relationship is confirmed, then health policies should be made to reduce the burden of RA.

## Abbreviations

25-OH-D: 25-hydroxycholecalciferol
1,25(OH)_2_D_3_: 1,25-dihydroxyvitamin D_3_
ACPA: Anticyclic citrullinated protein/peptide antibody
BC: British Colombian study
COPD: Chronic obstructive pulmonary disease
EIRA: Epidemiological Investigation of Rheumatoid Arthritis
HAP: Household air pollution
HR: Hazard ratio
LMICs: Low-and-middle-income countries
NF-ƙB: Nuclear factor ƙappa B
NHS: Nurses’ health study
NO_2_: Nitrates
OR: Odds ratio
O_3_: Ozone
PM: Particulate matters
PM_0.1_: Particulate matter with an aerodynamic diameter less than 0.1 μ
PM_2.5_: Particulate matter with an aerodynamic diameter less than 2.5 μ
PM_10_: Particulate matter with an aerodynamic diameter less than 10 μ
RA: Rheumatoid arthritis
SARDs: Systemic autoimmune rheumatic diseases
SLE: Systemic lupus erythematosus
SO_2_: Sulphur dioxide
Th1: T helper lymphocyte type 1
UVB: Ultraviolet B radiations
VDR: Vitamin D receptor
